# EZR–ROS1 rearrangement as a novel mechanism of acquired resistance to EGFR-TKIs in NSCLC: a case report and literature review

**DOI:** 10.3389/fimmu.2026.1758000

**Published:** 2026-01-26

**Authors:** Jia Hu, Fu Hui, Yiqing Jiang, Yi Zhang, Luxin Li, Mingxin Yu, Yue Chen, Yanhong Ding, Xuede Zhang

**Affiliations:** 1 Weifang People’s Hospital, Shandong Second Medical University, Weifang, China; 2 Oncology Laboratory of the First Affiliated Hospital, Weifang People’s Hospital, Shandong Second Medical University, Weifang, China; 3 Department of oncology, Weifang People’s Hospital, Shandong Second Medical University, Weifang, China

**Keywords:** case report, EGFR mutation, molecular targeted therapy, NSCLC, ROS1 rearrangement

## Abstract

ROS1 rearrangement is a rare mechanism of acquired resistance to epidermal growth factor receptor tyrosine kinase inhibitors (EGFR-TKIs), with an incidence of less than 1% in non-small cell lung cancer (NSCLC). However, the clinical characteristics and therapeutic strategies for patients who develop ROS1 rearrangement after resistance to EGFR-TKIs remain undefined. Here, we describe the first case of EGFR-TKIs resistance caused by the EZR exon 10–ROS1 exon 34 rearrangement. This case highlights ROS1 rearrangement as a rare but targetable mechanism of acquired resistance to EGFR-TKIs. Additionally, we conducted a comprehensive review of previously reported cases of other ROS1 rearrangements occurring after EGFR-TKIs resistance in NSCLC. Our analysis reveals that this rare mutation shares notable clinical similarities with primary ROS1 rearrangement in certain characteristics. However, it exhibits significant differences in fusion partner distribution and co-mutation frequency compared to the primary ROS1 rearrangement. The efficacy of crizotinib in this molecular subset demonstrates favorable clinical outcomes. Furthermore, considering the relatively high prevalence of ROS1 co-mutations with other genetic alterations in these cases, multi-targeted combination therapy may represent a promising therapeutic strategy for this distinct patient population.

## 1 Introduction

Non-small cell lung cancer (NSCLC) remains the leading cause of cancer-related deaths worldwide (1). Among its major driver mutations, epidermal growth factor receptor (EGFR) mutation and ROS proto-oncogene 1 (ROS1) rearrangements are mutually exclusive in NSCLC (2). However, ROS1 rearrangements have been reported as acquired alterations in some patients following EGFR tyrosine kinase inhibitors (EGFR-TKIs), suggesting a potential resistance mechanism to EGFR-targeted therapy. ROS1 rearrangements induce autophosphorylation, thereby activating downstream signaling pathways shared with EGFR and producing cellular phenotypes that resemble EGFR pathway activation, which may reduce sensitivity to EGFR-TKIs (3). Although individual case reports are available, systematic analyses of such patients remain scarce. Identifying bypass resistance mechanisms may provide clinical benefit by enabling more precise selection of subsequent targeted and combination therapies, thereby prolonging disease control and improving patient outcomes. Here, we report a novel case of long-term NSCLC survival exhibiting acquired resistance to EGFR-TKIs, in which ROS1 rearrangement emerged following third-generation TKI therapy without evidence of persistent EGFR mutation. Furthermore, we reviewed reported cases of ROS1 rearrangements emerging after EGFR-TKIs resistance, aiming to explore the clinical characteristics of this rare patient population and guide therapeutic strategies.

## 2 Case description

**Figure 1:**
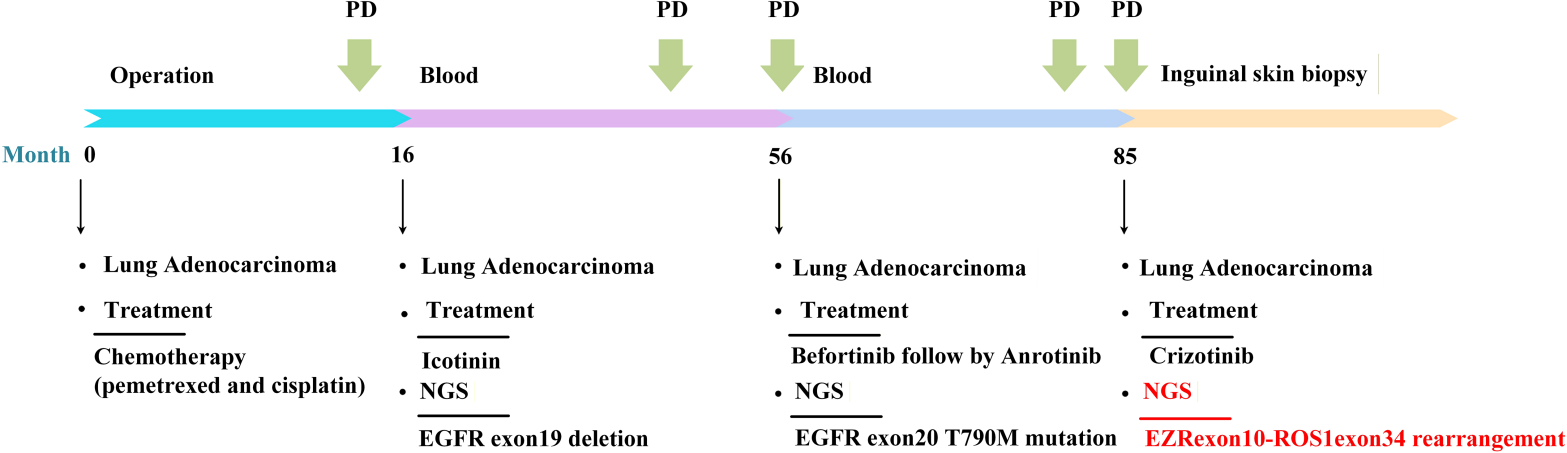
Timeline of major therapeutic interventions administered to the patient.

A 57-year-old female non-smoker with cough, sputum, and hemoptysis in sputum for a month was admitted to our hospital in September 2016 (**Figure 1**). She underwent thoracoscopic right lower lobectomy with mediastinal lymph node dissection. Postoperative histopathology confirmed invasive lung adenocarcinoma (TNM staging, 7th edition: pT2aN0M0, stage IB). The patient received one cycle of adjuvant chemotherapy with pemetrexed plus cisplatin, which was discontinued due to severe gastrointestinal toxicity.

In August 2017, she reported soreness and limited mobility in her left lower limb. Bone scintigraphy (BS) revealed abnormal radiotracer uptake in the distal femur, consistent with bone metastasis. She received conformal radiotherapy to the femoral lesion (40 Gy in 20 fractions), which effectively alleviated the pain. Subsequently, the patient received zoledronic acid until September 2019.

**Figure 2:**
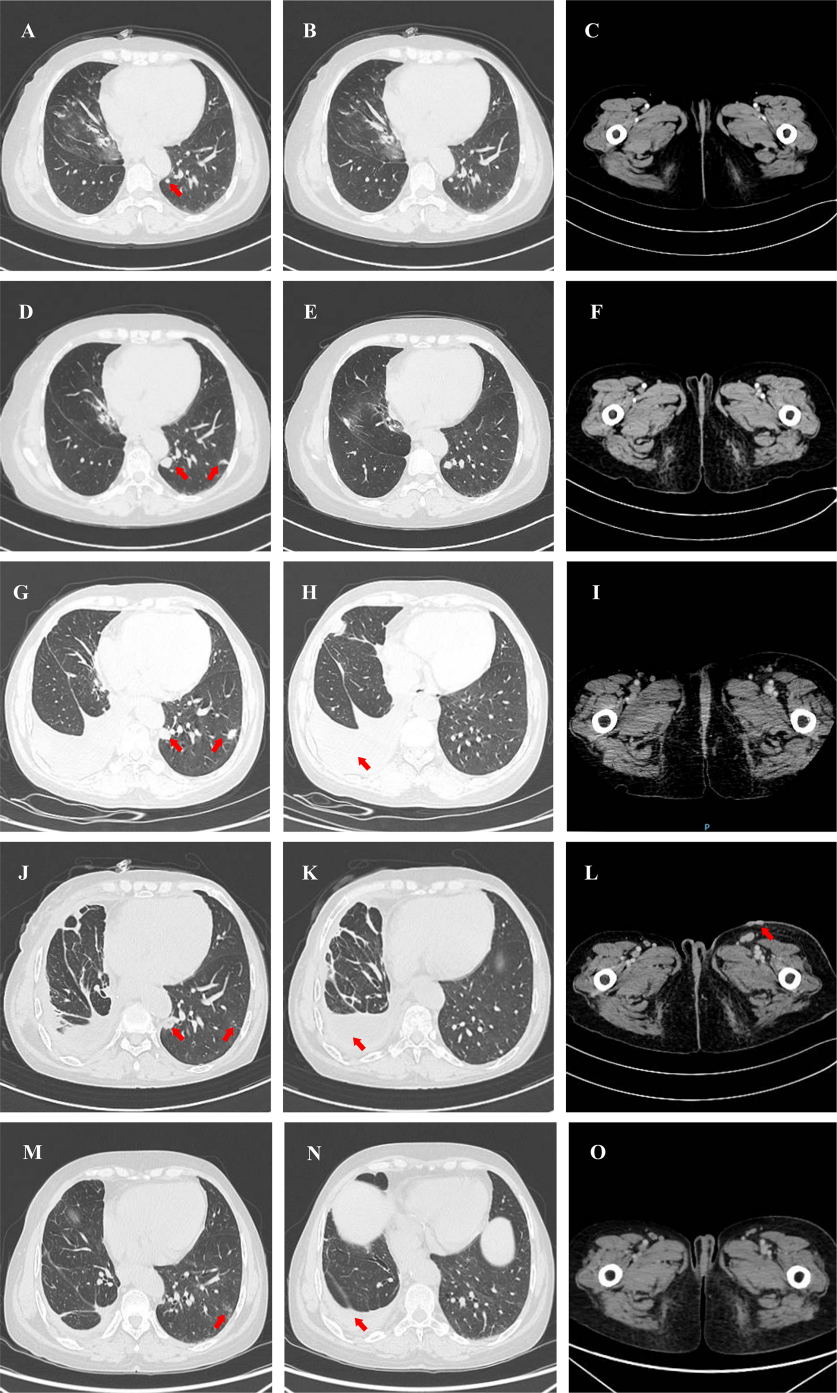
CT images of pulmonary tumor, pleural effusion, and inguinal region during treatment. **(A)**New pulmonary tumor after 23 months of icotinib. **(B)** No pleural effusion after 23 months of icotinib. **(C)** No abnormalities in the inguinal region after 23 months of icotinib. **(D)** Pulmonary tumor after 33 months of icotinib. **(E)** Pleural effusion after 33 months of icotinib. **(F)** No abnormalities in the inguinal region after 33 months of icotinib. **(G)** Pulmonary tumor after 31 months of befotertinib. **(H)** Pleural effusion after 31 months of befotertinib. **(I)** Inguinal region after 31 months of befotertinib. **(J)** Pulmonary tumor after 2 months of anlotinib. **(K)** Pleural effusion after 2 months of anlotinib. **(L)** Inguinal region after 2 months of anlotinib. **(M)** Pulmonary tumor after 21 months of crizotinib. **(N)** Pleural effusion after 21 months of crizotinib. **(O)** No abnormalities in the inguinal region after 21 months of crizotinib.

In December 2017, next-generation sequencing (NGS) revealed the presence of EGFR exon 19 deletion (EGFR 19del), and the patient was initiated on first-line targeted therapy with icotinib. In September 2019, BS indicated progression of the metastatic lesion in the distal left femur. Accordingly, incadronate (5 mg) was administered until January 2, 2020; treatment for the bone metastasis was subsequently discontinued at the patient’s request. In November 2019, follow-up computed tomography (CT) identified new metastatic lesions in the left lung, suggesting disease progression (**Figures 2A–C**). Given the limited extent of disease, the patient continued targeted therapy with close follow-up.

**Figure 3:**
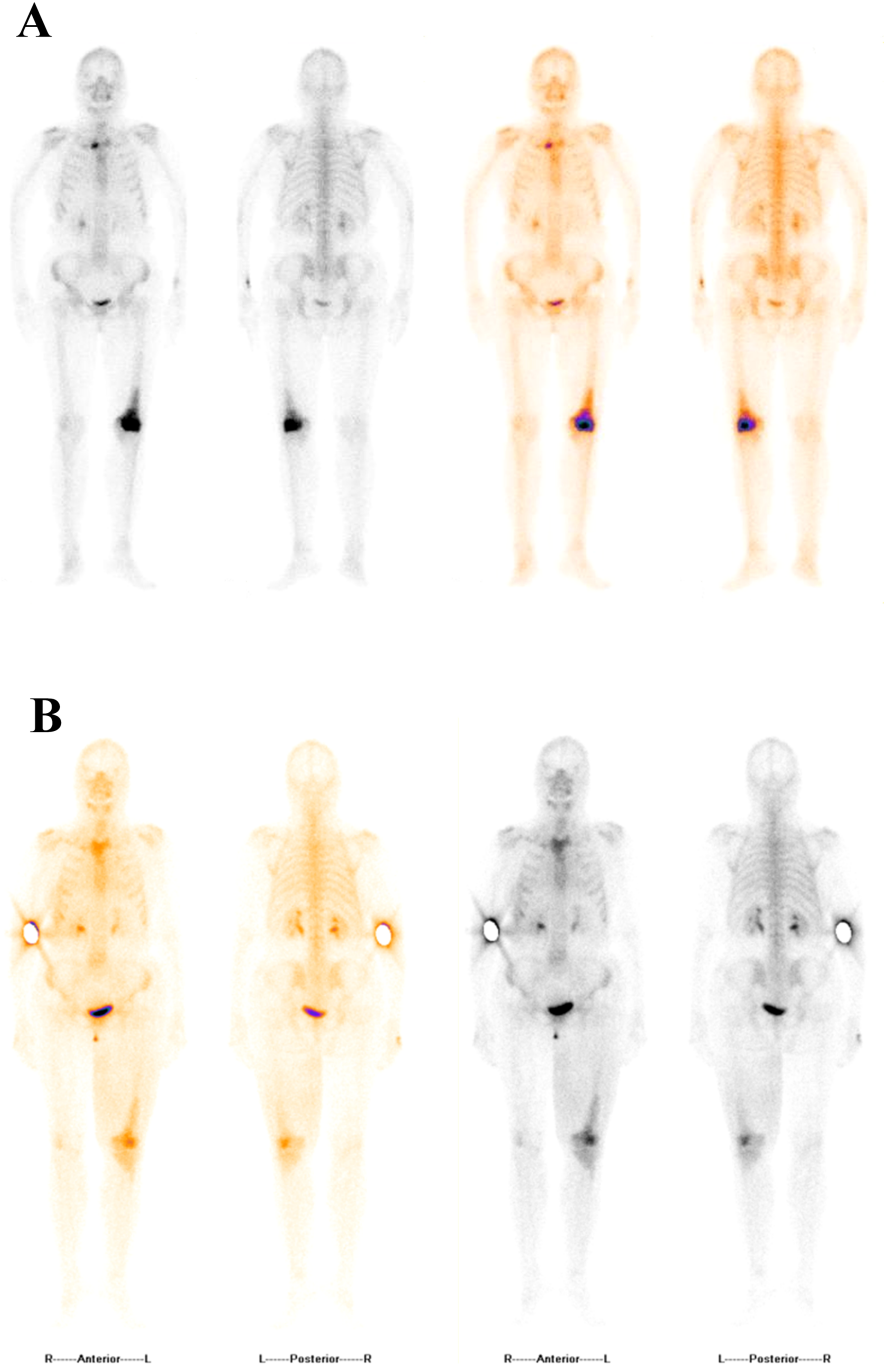
Serial bone scintigraphy demonstrated abnormal radiotracer uptake in the distal left femur and proximal left tibia, consistent with known bone metastases. Compared with 2021, the 2025 follow-up scan showed no new lesions and an overall decrease in uptake intensity, indicating stable bone metastatic disease. **(A)** December 31, 2021. **(B)** September 1, 2025.

In the follow-up, CT (**Figures 2D–F**) performed on September 30, 2020, indicated an increase in the size of the tumor in the left lower lobe of the lung, accompanied by a small amount of pleural effusion in the right lung. From December 24, 2020 to March 18, 2021, the patient received MW032/Xgeva (denosumab) (120 mg) for a total of four doses. From May 27, 2021 to the present, she has been receiving incadronate disodium (10 mg). In December 2021, follow-up BS was performed (**Figure 3A**). In April 2021, a repeat NGS revealed the presence of an acquired EGFR T790M mutation in exon 20. As a result, the patient initiated second-line therapy with befotertinib, and the therapeutic evaluation at 31 months indicated stable disease (**Figures 2G–I**).

In June 2023, the patient reported chest tightness. One month later, pulmonary artery computed tomography angiography revealed a right-sided pulmonary embolism accompanied by massive pleural effusion. Given the patient’s severe gastrointestinal toxicity after the first cycle of chemotherapy, a low-dose intrapleural instillation of cisplatin (30 mg) in combination with endostatin (45 mg) was administered. Prophylactic antiemetic therapy was provided with palonosetron hydrochloride injection (0.25 mg) and metoclopramide hydrochloride (10 mg) to prevent gastrointestinal adverse effects. Fortunately, this treatment resulted in marked symptomatic relief, and no significant gastrointestinal toxicity was observed. In August 2023, third-line therapy with anlotinib was initiated.

During this period, the patient noted progressive enlargement of a left inguinal mass. A follow-up CT (**Figures 2J–L**) in October 2023 revealed multiple enlarged lymph nodes in the left inguinal region with associated skin thickening, suggestive of disease progression. A subsequent biopsy of the skin lesion confirmed metastatic adenocarcinoma of pulmonary origin. NGS of the biopsy specimen identified a ROS1 rearrangement (EZR exon 10–ROS1 exon 34). NGS was performed using a commercially available human EGFR/KRAS/BRAF/PIK3CA/ALK/ROS1 mutation detection kit (reversible terminator sequencing; Yuanma Gene Technology Co., Ltd., China; NMPA registration No. 20213400525) on an Illumina MiniSeq platform (USA), with a sequencing depth of ≥500×. The variant allele frequency was 0.95%. In November 2023, fourth-line treatment with crizotinib was initiated. On August 27, 2025, treatment response evaluation showed a partial response (PR) (**Figures 2M–O**). On September 1, 2025, follow-up BS indicated stable lesions (**Figure 3B**). The only reported adverse event was moderate lower limb edema, with no other intolerable side effects, indicating good overall tolerability.

## 3 Discussion

The EGFR gene, located on chromosome 7p11.2, encodes a transmembrane receptor tyrosine kinase belonging to the ErbB family, which plays a pivotal role in regulating cellular proliferation, differentiation, migration, and apoptosis (4). EGFR alterations are most frequently identified in non-smoking females of East Asian descent with lung adenocarcinoma (5). Two classical activating mutations—EGFR 19del and EGFR leucine-to-arginine substitution at codon 858(EGFR L858R) —account for approximately 85% of all EGFR mutations in NSCLC (6).

The advent of EGFR-TKIs has revolutionized the treatment of EGFR-mutant NSCLC, yet acquired resistance remains a major clinical challenge. The molecular mechanisms underlying acquired resistance are highly complex and heterogeneous, and can be broadly classified into three categories: EGFR pathway reactivation, EGFR pathway bypass, and EGFR pathway indifference (7, 8). Among resistance mechanisms, the secondary EGFR mutation T790M is particularly critical. As a canonical “gatekeeper” mutation, T790M increases ATP binding affinity, thereby reducing the potency of ATP-competitive kinase inhibitors (9). The EGFR pathway bypass involves the activation of parallel signaling pathways that re-engage the downstream oncogenic transcriptional output of the original EGFR pathway. These alternative oncogenic drivers, primarily RTKs other than EGFR, can be activated through mechanisms such as fusions (e.g., ROS1), thereby reactivating downstream signaling and driving the development of resistance (10).

The ROS1 gene, located at chromosome 6q22, encodes a receptor tyrosine kinase within the insulin receptor family (11). ROS1 rearrangements are more frequently observed in young subjects, women and never-smokers (12). ROS1 rearrangements arise from chromosomal breakage and non-homologous end joining, typically involving the 3′ region of ROS1—encompassing its C-terminal kinase domain—and the 5′ end of various partner genes (11). The most common breakpoints are found within introns 31 to 35, with intron 31 serving as the canonical site of disruption (13, 14). The most prevalent fusion partner is CD74, accounting for approximately 40% of ROS1 rearrangements. Its membrane localization signal is believed to enhance the stability of the resulting fusion protein. The second most common partner is EZR (10%–20%), which encodes ezrin-a protein with intrinsic dimerization capability that facilitates downstream signaling. In addition, more than 50 other fusion partners have been identified, including SDC4, TPM3, and SLC34A2, though each occurs less frequently (13–15). The prognostic implications of different ROS1 rearrangements remain controversial. Some studies have reported significantly longer progression-free survival (PFS) and overall survival (OS) in patients with non-CD74–ROS1 rearrangements compared to those with CD74–ROS1 rearrangements (16). However, other investigations have failed to demonstrate a correlation between fusion partners and clinical outcomes (13). Additionally, no significant differences have been observed in the breakpoints located within various ROS1 introns among fusion variants (13).

Crizotinib was the first agent approved for treating patients with ROS1-rearranged NSCLC. A study demonstrated that, in patients with ROS1-positive NSCLC, crizotinib achieved an objective response rate of 71.7% and a median progression-free survival of 15.9 months (clinical trial information: NCT01945021) (17).

**Figure 4:**
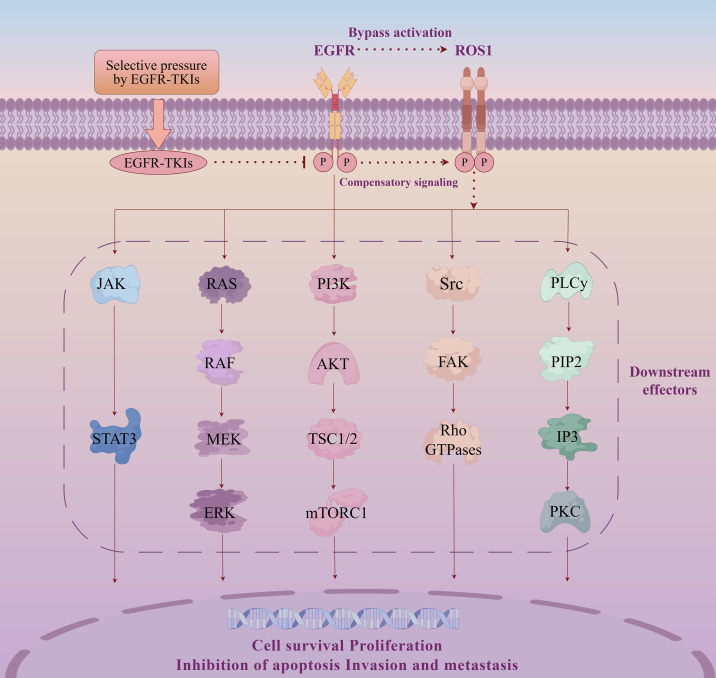
ROS1-mediated bypass signaling under EGFR-TKIs pressure. EGFR and ROS1 are receptor tyrosine kinases that converge on overlapping downstream pathways, including JAK–STAT, RAS–ERK, PI3K–AKT–mTOR, Src–FAK–Rho GTPase, and PLCγ–PKC signaling. Although EGFR-TKIs suppress EGFR phosphorylation, ROS1 rearrangement may act as a compensatory bypass driver to sustain oncogenic signaling outputs. This convergence promotes continued tumor cell survival and proliferation and may facilitate invasion and metastasis, providing a mechanistic rationale for considering ROS1-targeted strategies in selected EGFR-TKI–resistant tumors.

EGFR mutations and ROS1 rearrangements are both key oncogenic drivers in NSCLC and are generally considered mutually exclusive (2). However, with the widespread adoption of NGS, increasing evidence suggests that EGFR mutations and ROS1 rearrangements can coexist (18). Among EGFR-mutant NSCLC patients undergoing EGFR-TKIs therapy, the prevalence of concurrent ROS1 rearrangements is less than 1%. This secondary ROS1 rearrangement can present either as mutually exclusive with EGFR mutations or as a concomitant alteration. The mechanisms by which ROS1 rearrangements confer resistance to EGFR-TKIs remain incompletely understood. Evidence suggests that EGFR-TKIs suppress EGFR autophosphorylation and downstream signaling pathways, thereby inducing tumor regression in EGFR-dependent clones (19). Under sustained drug pressure, tumors may escape by restoring these same downstream signaling outputs through the activation of non-EGFR kinases. As a mechanism of EGFR pathway reactivation, ROS1 rearrangements can induce autophosphorylation and sustained signaling, leading to the activation of downstream transcription factors shared with EGFR (11). Evidence suggests that EGFR, upon binding its ligand, undergoes a conformational change, leading to receptor dimerization/oligomerization and phosphorylation at its C-terminus (3). Subsequently, phosphorylated tyrosine residues activate docking sites for downstream effectors, including the PI3K/AKT, Ras/Raf/MEK/ERK, JAK/STAT, and PLCγ/PKC pathways (3) (**Figure 4**). This results in cellular phenotypes that resemble EGFR pathway activation, rendering tumor cells less sensitive to EGFR-TKIs and ultimately contributing to the development of EGFR-TKI resistance.

Cases of ROS1 rearrangement following acquired resistance to EGFR-TKIs in NSCLC patients are exceedingly rare. To our knowledge, this is the first reported case of EGFR-TKIs resistance associated with the EZRexon10–ROS1exon34 rearrangement. We performed a structured literature search to identify published evidence on ROS1 rearrangement emerging as an acquired resistance mechanism to EGFR-TKIs in NSCLC. We searched PubMed from database inception to 2025-11-30. The search terms were “EGFR” AND”ROS1”, and results were restricted to the publication type case report. Titles and abstracts were screened, followed by full-text review of potentially relevant records. We included reports of NSCLC patients treated with EGFR-TKIs in whom ROS1 rearrangement was identified as a putative acquired resistance mechanism. Additional relevant papers were identified by screening the reference lists of included articles. To the best of our knowledge, within the scope of our search, the included reports represent all identifiable published cases. A review of the literature identified only seven reported cases in which EGFR-TKI resistance was associated with ROS1 rearrangements (**Table 1**).

**Table 1:** A summary of cases in which ROS1 rearrangement contributes to resistance to EGFR-TKIs.

Study	Origin	Gender	Smoking status	Primary genetic mutations	Medication following genetic mutation	Subsequent genetic mutations	Medication after ROS1 Rearrangement	Adverse events	Best response	PFS	OS
Zeng L.et al (20)	China	Female	Never-smoker	EGFR 19del	Icotinib → osimertinib	EGFR 19del GOPC–ROS1 rearrangement	Crizotinib and osimertinib	Grade 2 rash and diarrhea	PR	NR	NR
Xu S.et al (21)	China	Female	NR	EGFR L858R	Gefitinib → AP → afatinib → DP	ROS1–ADGRG6 rearrangement	Crizotinib	None	SD	7m	NR
Qiu D. et al (22)	China	Male	Smoker	EGFR L858R	gefitinib	RGFR L858R ROS1 fusion	AP	Grade 2 hematologic and gastrointestinal	CR	2y+	2y+
Zhu Y.-C. et all (23)	China	Female	Never-smoker	EGFR 19del	gefitinib → pemetrexed → AP	SDC4–ROS1 rearrangement	Crizotinib	Grade 1 rash	PR	16m	NR
Xu C. et all (24)	China	Male	Never-smoker	EGFR L858R	AP → icotinib → osimertinib	EGFR L858R MET amplification TPD52L1–ROS1 rearrangement	Crizotinib and osimertinib	Severe gastrointestinal toxicity	PR	NR	NR
Zhang S. et all (25)	China	Male	Never-smoker	EGFR L858R	Gefitinib	EGFR L858R EGFR T790M OPRM1-ROS1 rearrangement	Osimertinib	NA	PD	7m	1y
Plomer E. et all (26)	Switzerland	Female	Never-smoker	EGFR L858R	Erlotinib → AP	EGFR L858R HER2 mutation MET amplification ROS1 fusion	Crizotinib and afatinib	Stable lesions	PR	17m	4y+
Present case	China	Female	Never-Smoker	EGFR 19del	Icotinib → befotertinib → Anrotinib	EZR–ROS1 rearrangement	Crizotinib	Lower limb edema	PR	23m	23m

NR, not reported; AP, pemetrexed and cisplatin; DP, docetaxel and carboplatin; SD, stable disease; CR, complete response; PR, partial response.

A retrospective analysis of these cases revealed several shared clinical features: adenocarcinoma histology (8/8), Asian ethnicity (7/8), never-smokers (6/8) and female (5/8). This is highly similar to primary ROS1 rearrangements.

All patients initially carried classical activating mutations, with EGFR L858R being the most frequent (5/8), followed by EGFR 19del (3/8). First-generation EGFR-TKIs were administered following detection of EGFR mutations. Upon disease progression, ROS1 rearrangements were detected either in the second round of genetic testing (5/8), or in a third round after the second test revealed only additional EGFR mutations (3/8). Intriguingly, three patients received icotinib as initial therapy and subsequently developed EGFR T790M mutations, retaining the original activating mutations across serial tests, thereby demonstrating co-mutation status. In contrast, our case exhibited a distinct evolutionary trajectory, with complete loss of the initial EGFR 19del in the second biopsy and retention of only T790M, followed by detection of a solitary ROS1 rearrangement in the third test.

Notably, in cases where ROS1 rearrangements occur after TKI resistance, the probability of co-mutation with other genes is significantly increased. At the final genetic testing, most patients (5/8) exhibited co-mutations involving both EGFR and ROS1, while a minority (3/8) showed loss of the original EGFR mutation. Among the six patients with identified fusion partners, the ROS1 rearrangements involved different partner genes, which are unrelated to the common distribution of fusion sites seen in initial ROS1 rearrangements. This unique resistance mechanism has been observed in all three generations of EGFR-TKIs. We hypothesize that this may represent a resistance mechanism shared across EGFR-TKIs; however, the underlying molecular pathways remain incompletely understood and warrant further investigation.

Upon detection of ROS1 rearrangement, most reported patients received crizotinib. In this limited series, the objective response rate was 75% and the median progression-free survival was 16.5 months, appearing broadly similar to outcomes reported for primary ROS1-rearranged NSCLC. Combination therapy with crizotinib and osimertinib has shown clinical benefit in patients harboring concurrent co-mutations of EGFR and ROS1. Notably, a patient with this characteristic achieved a complete response after treatment with pemetrexed plus cisplatin, with PFS exceeding 24 months. This finding suggests that conventional chemotherapy may remain a viable therapeutic option within a personalized treatment strategy. According to the NCCN Guidelines, entrectinib, crizotinib, repotrectinib, and ceritinib are recommended treatment options in the first-line setting for patients with ROS1 fusion–positive NSCLC (27). In a subset of tumors with acquired resistance, the original EGFR mutation is retained while an acquired ROS1 fusion emerges as a parallel oncogenic driver. In this context, dual blockade (EGFR-TKI plus a ROS1 inhibitor) is mechanistically plausible, as EGFR and ROS1-driven signaling may differentially contribute to pathway output and intratumoral heterogeneity. Conversely, in some post-resistance biopsy specimens, the EGFR mutation signal is markedly attenuated or no longer detectable, which appears consistent with a shift from EGFR dependence to an acquired fusion as the dominant oncogenic driver. For patients who have received multiple lines of TKIs or are not candidates for further targeted therapy, systemic chemotherapy, with or without an anti-angiogenic agent, remains a commonly considered approach; pemetrexed-based regimens are frequently used as an important backbone in clinical practice (27, 28). Finally, for ROS1 fusion–driven NSCLC, the overall benefit of ICI monotherapy appears limited, and treatment decisions are often made after an individualized assessment, frequently in the context of chemotherapy-based strategies (28). Further investigation into the molecular interaction between EGFR and ROS1 pathways may uncover rational combination strategies.

## 4 Conclusion

This study reports the first case of EGFR-TKIs resistance in a NSCLC patient caused by an EZRexon10–ROS1exon34 rearrangement, providing new insights into EGFR-TKIs resistance research. Compared to primary ROS1 rearrangements, resistance mutation exhibits a dispersed distribution of fusion partners and a higher frequency of co-mutations, while demonstrating similarities in clinical features, such as demographic characteristics. Crizotinib was associated with clinically meaningful responses in this small cohort. Furthermore, further investigation of the molecular interactions between the EGFR and ROS1 pathways is recommended, along with the exploration of multi-target combination therapy strategies.

## Data Availability

The original contributions presented in the study are included in the article/supplementary material. Further inquiries can be directed to the corresponding authors.
